# Long-term Efficacy of Insulin Pump Therapy in Children with Type 1 Diabetes Mellitus

**DOI:** 10.4274/Jcrpe.751

**Published:** 2012-09-11

**Authors:** Ruby Joshi Batajoo1, Catherine R. Messina, Thomas A. Wilson

**Affiliations:** 1 Stony Brook University, Department of Pediatrics, Stony Brook, New York, USA; 2 Stony Brook University, Department of Preventive Medicine, Stony Brook, New York, USA; 3 Stony Brook University, Department of Pediatrics, Division of Pediatric Endocrinology, Stony Brook, New York, USA

**Keywords:** diabetes mellitus, glycated hemoglobin, hemoglobin A1c, insulin pump, diabetic control, long term efficacy, continuous subcutaneous insulin infusion

## Abstract

**Objective:** Insulin pumps have been well established for insulin delivery. However, questions about long-term efficacy of insulin pump therapy still remain. We evaluated the long-term efficacy of continuous insulin infusion pump therapy (CSII) in pediatric patients with type 1 diabetes mellitus (T1DM).

**Methods:** This was a retrospective observational study which included 131 patients with T1DM who transitioned to an insulin pump from multiple daily insulin (MDI) injections between 1999 and 2009 and were followed by one endocrinologist. Data were collected from 6 months prior to switching to CSII to 30 months after initiation of CSII and included glycated hemoglobin (HbA1c) and insulin requirement. Of the 131 patients, 45 had complete data consisting of a visit and HbA1c every 6 months for 30 months after transition to CSII and were included for analysis.

**Results:** Mean HbA1c prior to starting the CSII was 8.0 +0.9 %, 7.7 +1.0 % at 6 months and 7.8+1.2 % at 1 yr post initiation of CSII. However, at 30 months, HbA1c increased to 8.0+1.3%. A trend in transient improvement in HbA1c was limited only to those patients >11 yr of age and those requiring >0.75 u/kg/day of insulin at transition and was not seen in those <11 yr of age or those requiring <0.75 u/kg/day and did not persist beyond 1 year.

**Conclusions:** There was no long-term significant difference in glycemic control in patients with CSII as compared to MDI.

**Conflict of interest:**None declared.

## INTRODUCTION

The Diabetes Control and Complications Trial demonstrated that intensive diabetes control during childhood significantly reduces the microvascular complications (1). Since the inception of continuous insulin infusion by insulin pumps (CSII) in the 1970s, the popularity of CSII has been increasing (2). CSII is intensive insulin therapy which attempts to mimic physiological insulin release by administration of 24-hour adjustable basal rates and flexible mealtime bolus doses (3). Many studies have been done comparing CSII with multiple daily insulin (MDI) injections as regards to its efficacy and safety. Overall metabolic control was found to be similar in some studies (2,4,5,6,7). Other studies (8,9,10,11,12,13,14,15) found better glycemic control with CSII. In adults, Reznik et al (16) found that CSII was effective, particularly in patients with baseline glycated hemoglobin (HbA1c) of above 8% and may persist until 6-yr follow-up. However, long-term follow-up studies regarding diabetes control in children with CSII are limited.

Our study was designed to evaluate the long-term diabetic control of children with type 1 diabetes mellitus (T1DM) who had transitioned to an insulin pump.

## METHODS

This was a retrospective study of patients with T1DM followed by one pediatric endocrinologist at Stony Brook University Medical Center who had been transitioned to CSII between 1999 and 2009. Data were collected by reviewing charts and computer flowsheets over a period of 36 months (six months prior to starting CSII to 30 months post transition to CSII). Generally, patients were asked to return at 3-4-month intervals with HbA1c determinations obtained in commercial laboratories dictated by their insurance carrier prior to the visit. Data collected included: age, sex, age of onset of T1DM, age at transition to CSII, HbA1c, height, weight, body mass index (BMI) and insulin dose. 

131 patients were initially identified who had transitioned to CSII. Of these, 45 patients had complete data as defined by a visit and HbA1c at least every 6 months for the 36-month period; these patients comprise the analysis sample. 

Descriptive statistics (means, standard deviations, frequencies and proportions) were obtained for all study variables. Continuous data were assessed for departures from the normal distribution using the Shapiro-Wilk test of normality. When distributions approximated the normal curve, parametric tests were employed; non-parametric alternatives were utilized when data were not normally distributed. Between-subject bivariate comparisons (displayed in Table 1) were conducted using the chi-square test of association for categorical variables and the independent samples t-test (or Wilcoxon-Mann-Whitney test) for continuous data. 

Repeated measures analysis of variance (ANOVA) or the non-parametric Friedman test were used to examine overall changes in mean values for HbA1c and insulin requirement over time (degrees of freedom for ANOVAs were corrected whenever Mauchly’s test indicated that the assumption of sphericity was violated). These were followed up with paired samples t-tests (or Wilcoxon signed-ranks test) to explore comparisons of clinical values at specific time points. We similarly conducted 2-way repeated measures ANOVAs to explore the effects of adolescence (<=11 years of age vs. >11 years of age) and median insulin requirement at transition to a pump (>0.75 u/kg/day vs. <=0.75 u/kg/day) on diabetic control. 

All tests of significance were two-sided and evaluated at the p<0.05 level. Based on recommendations by Rothman and Streiner & Norman (17,18), p-values were not adjusted for multiple comparisons because “family wise” comparisons were not conducted. That is, only two groups were compared for the age group and insulin requirement comparisons (e.g., patient age at transition: <=11 years of age vs. >11 years of age; and insulin requirement at transition: >0.75 u/kg/day vs. <=0.75 u/kg/day). Post-hoc comparisons were conducted only when the ANOVA F-test was significant. Only one outcome was examined: long-term diabetic control (as measured by HbA1c) of children with T1DM who had transitioned to an insulin pump. 

Data analyses were conducted using SPSS Statistics (Version 19, IBM SPSS Statistics, Somers, NY). This project received Institutional Review Board approval.

## RESULTS

Demographics of the study population are described in Table 1. 131 patients were included in the study. Overall, 72 (54.5 %) were male and 59 (45 %) were female. Mean age of onset of DM was 7.2+3.9 years. As shown in Table 1, demographic characteristics of patients followed up for 30 months post transition did not differ significantly from patients without complete follow-up. Thus, only the 45 patients who were followed up for 30 months post transition were included in the final analysis. However, compared to these patients with complete data, patients with incomplete data for 30 months post transition to CSII had higher baseline HbA1c (p=0.01, Table 1). 

**Figure 1:**
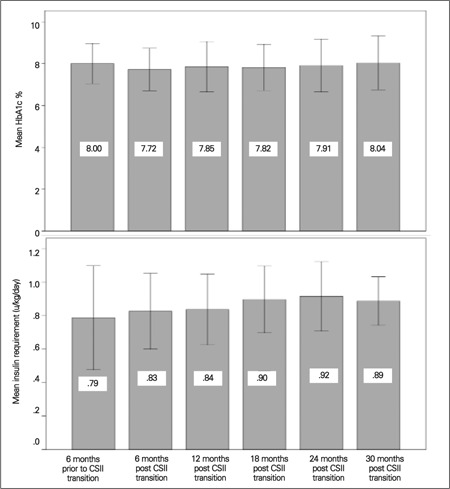
Mean HbA1c% and insulin requirement over 30-month follow-up (n = 45 children)

Overall, no significant effect for time and HbA1c emerged [F (4.5,198.4) = 1.28, p = 0.28; degrees of freedom corrected using Huynh-Feldt estimates of sphericity (ε=0.90)] although the findings suggested a marginal quadratic component [F(1, 44)=3.78, p<0.06]. The mean HbA1c on multiple dose insulin was 8.0+0.9% which appeared to improve to 7.7+1.0 % at 6 months post transition to pump. However, this improvement did not persist and by 30 months, HbA1c was back to 8.0+1.3 % despite a significant progressive linear increase in insulin requirement (also shown in Figure 1; [F (2.8, 104.4) = 3.90, p=0.01; degrees of freedom corrected using Greenhouse-Geisser estimates of sphericity (ε=0.55)]; linear component, [F (1,38) = 6.31, p = 0.02]. 

**Figure 2:**
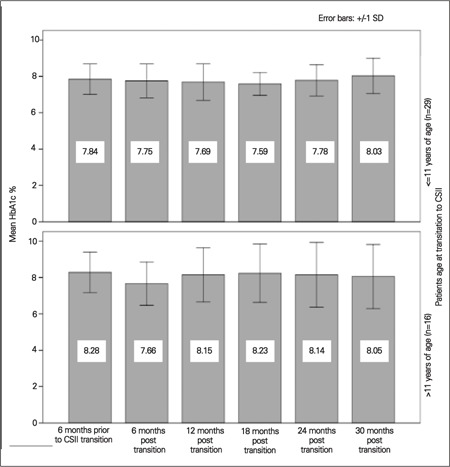
HbA1c% over 30 months by patient age at transition to CSII

We next conducted a two-way repeated measures ANOVA including age group as a between-subjects factor. We defined age group as those ≤11 and >11 years of age based on approximate transition into adolescence to explore the impact of adolescence on diabetic control. HbA1c after transition to CSII in these age groups is shown in Figure 2. No significant effects of time by age group were detected [F (4.5, 193.9) =1.69, p=0.14; degrees of freedom corrected using Huynh-Feldt estimates of sphericity (ε=0.90)]. In patients ≤11 yr, no difference in HbA1c is seen at 6 months of transition to a pump as compared to those >11 yr where the HbA1c appeared to drop by 0.6% 6 months after transition). However, this improvement did not persist and returned to 8.2+1.6% by 18 months. 

**Figure 3:**
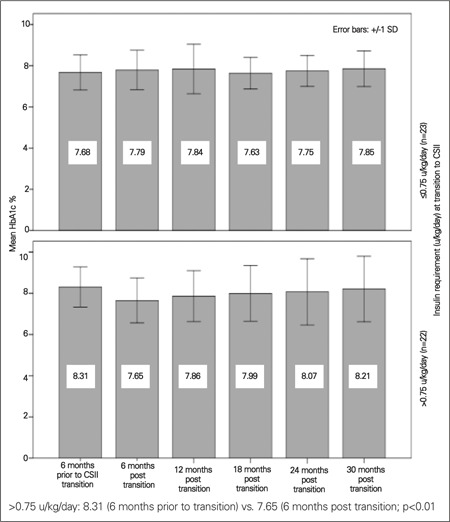
HbA1c% over 30-month follow-up by insulin requirement at transition to CSII

Our final two-way repeated measures ANOVA included median insulin requirement at transition to a pump as a between-subjects factor. 22 patients required ≤0.75 u/kg/day and 23 patients required >0.75 u/kg/day at transition to a pump. A significant effect of time was detected among those requiring more insulin only [F (3.9, 86.3) = 2.97, p = 0.045; degrees of freedom corrected using Huynh-Feldt estimates of sphericity (ε=0.78)] which included a significant quadratic component [F(1, 22) =9.38, p<0.01]. Those requiring more insulin at transition demonstrated a transient improvement in HbA1c seen at 6 months after transition (p<0.01), whereas those requiring less insulin did not (Figure 3); this improvement did not persist either. 

## DISCUSSION

HbA1c is an objective measure of average blood glucose concentration over approximately the previous 2 months. Although the ideal goal is to achieve an HbA1c value as close to normal as possible, recommended goals vary with age: 7.5% to 8.5% for toddlers and preschoolers (<6 yr), less than 8% for school-age children (6 to 12 yr), and less than 7.5% for adolescents and young adults (13 to 19 yr) (19).

Although the demographics were comparable, baseline HbA1c in the 45 patients with complete data was significantly lower than in the remaining 86 patients with incomplete data. It is possible that the patients with complete data were more likely to comply with follow-up visits and laboratory tests. 

Many studies have been conducted to compare the efficacy of MDI versus CSII. In a systemic review of 22 studies, Jeitler et al (20) found that CSII resulted in a greater reduction of HbA1c. Maniatis et al (13) also found similar results with a decrease in HbA1c and a reduction in hypoglycemic episodes. In a retrospective chart review, Berhe et al (8) found that CSII is safe, effective and a superior alternative to MDI. Other studies have shown no difference in the glycemic control in these two modalities of treatment (2,5,6,7). In a literature review, Fuld et al (21) reported that nonrandomized studies reported a consistent fall in HbA1c in the CSII group, while the same improvement was not seen in randomized studies. Our study found no statistically significant sustained difference in HbA1c pre and post insulin pump in our patients overall. However, patients >11 yr at transition to a pump were found to have statistically significant improvement in HbA1c by 0.6 % at 6 months post transition (Figure 2). This is a clinically significant result considering that Diabetes Control and Complication trial (1) has reported a 21-49% decreased risk for microvascular complications with every 1% decrease in HbA1c. 

Many studies have looked at glycemic control on CSII in children, but most studies lack comparison of different age groups. In a retrospective paired study of 279 patients, Nimri et al (9) divided the entire cohort into prepubertal (1.6-8.6 yr, median 5.4 yr), adolescent (9-17 yr, median 13.7 yr) and young adults (17-40 yr, median 22.8 yr). The young adult group had the most favorable response in HbA1c followed by prepubertal children and adolescents. We documented transient improvement only in those children >11 yr of age and in those requiring >0.75 units of insulin/kg/day. We speculate that this may be due either to enhanced transient parental supervision or to the novelty of a new treatment modality encouraging better compliance in the older age group. 

Our findings are consistent with a multicenter study by Jakisch et al (6) which analyzed 434 matched pairs and found HbA1c to be significantly lower in the first year of CSII but rose to the same level by year 3. Similarly, Plotnick et al (22) also found only transient improvement in HbA1c at 3-6 months after pump therapy. In contrast, one study in children with T1DM (9) and one study in adults with T2DM (16) reported long-term improvement in HbA1c after initiation of insulin pump therapy. 

It should be stressed, however, that although no long-term improvement in HgbA1c occurred in our population, the advantages of insulin pump therapy on quality of life make this form of insulin delivery attractive. In addition, we did not examine other complications of diabetes such as hypoglycemia or occurrences of diabetic ketoacidosis.

A limitation of this study is the small sample size which may not represent the general population of children with diabetes mellitus. The small sample size also reduced the power of our statistical analyses to detect significant differences where they really exist. Thus, we did not adjust p-values for multiple comparisons, which may increase the likelihood that some statistically significant comparisons were detected by chance. Also, confounding factors such as the influence of behavior, parental supervision and other comorbidities have not been objectively measured or controlled for in these analyses.

## CONCLUSION

Our study shows no continued long-term improvement in HbA1c of CSII over MDI. However, transient improvement is seen in the older age group and those requiring more insulin per day, while the younger age group and those with less insulin requirement had relatively stable HbA1c during the study period. Our study indicates that compliance and motivation may play more significant role as factors in diabetic control than different insulin regimens. Therefore, it is important to address these factors to patients and families while giving them a choice between different insulin regimes. 

## Figures and Tables

**Table 1:**
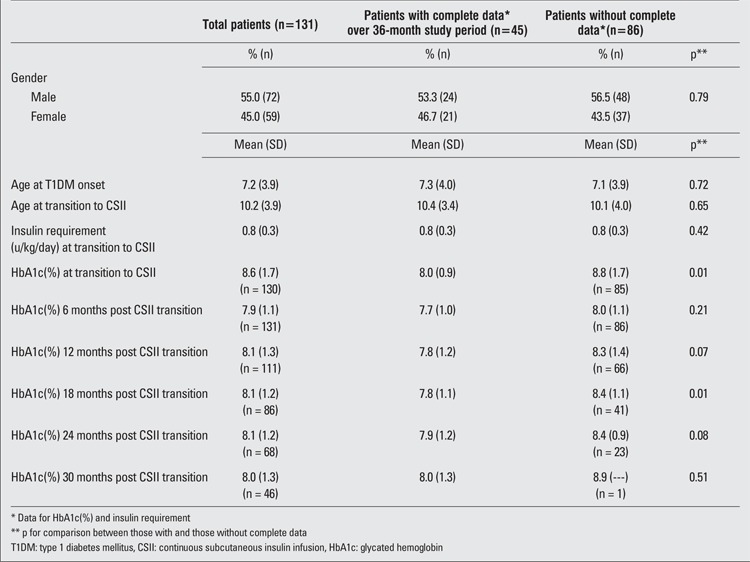
Characteristics of study sample

## References

[1] (1993). The effect of intensive treatment of diabetes on the development and progression of long-term complications in insulin-dependent diabetes mellitus. The Diabetes Control and Complications Trial Research Group. N Engl J Med.

[2] Wilson DM, Buckingham BA, Kunselman EL, Sullivan MM, Paguntalan HU, Gitelman SE (2005). A two-center randomized controlled feasibility trial of insulin pump therapy in young children with diabetes. Diabetes Care.

[3] Weinzimer SA, Ternand C, Howard C, Chang CT, Becker DJ, Laffel LM, Insulin Aspart Pediatric Pump Study Group (2008). A randomized trial comparing continuous subcutaneous insulin infusion of insulin aspart versus insulin lispro in children and adolescents with type 1 diabetes. Diabetes Care.

[4] Weintrob N, Benzaquen H, Galatzer A, Shalitin S, Lazar L, Fayman G, Lilos P, Dickerman Z, Phillip M (2003). Comparison of continuous subcutaneous insulin infusion and multiple daily injection regimens in children with type 1 diabetes: a randomized open crossover trial. Pediatrics.

[5] Fox LA, Buckloh LM, Smith SD, Wysocki T, Mauras N (2005). A randomized controlled trial of insulin pump therapy in young children with type 1 diabetes. Diabetes Care.

[6] Jakisch BI, Wagner VM, Heidtmann B, Lepler R, Holterhus PM, Kapellen TM, Vogel C, Rosenbauer J, Holl RW, German/Austrian DPV Initiative and Working Group for Paediatric Pump Therapy (2008). Comparison of continuous subcutaneous insulin infusion (CSII) and multiple daily injections (MDI) in paediatric Type 1 diabetes: a multicentre matched-pair cohort analysis over 3 years. Diabet Med.

[7] Nabhan ZM, Kreher NC, Greene DM, Eugster EA, Kronenberger W, DiMeglio LA (2009). A randomized prospective study of insulin pump vs. insulin injection therapy in very young children with type 1 diabetes: 12-month glycemic, BMI, and neurocognitive outcomes. Pediatr Diabetes.

[8] Berhe T, Postellon D, Wilson B, Stone R (2006). Feasibility and safety of insulin pump therapy in children aged 2 to 7 years with type 1 diabetes: a retrospective study. Pediatrics 2006;117:2132-2137.

[9] Nimri R, Weintrob N, Benzaquen H, Ofan R, Fayman G, Phillip M (2006). Insulin pump therapy in youth with type 1 diabetes: a retrospective paired study. Pediatrics.

[10] Doyle EA, Weinzimer SA, Steffen AT, Ahern JA, Vincent M, Tamborlane WV (2004). A randomized, prospective trial comparing the efficacy of continuous subcutaneous insulin infusion with multiple daily injections using insulin glargine. Diabetes Care.

[11] Sulli B, Shashaj B (2003). Continuous subcutaneous insulin infusion in children and adolescents with diabetes mellitus: decreased HbA1c with low risk of hypoglycemia. J Pediatr Endocrinol Metab.

[12] Juliusson PB, Graue M, Wentzel-Larsen T, Sovik O (2006). The impact of continuous subcutaneous insulin infusion on health-related quality of life in children and adolescents with type 1 diabetes. Acta Paediatr.

[13] Maniatis AK, Klingensmith GJ, Slover RH, Mowry CJ, Chase HP (2001). Continuous subcutaneous insulin infusion therapy for children and adolescents: an option for routine diabetes care. Pediatrics.

[14] Bin-Abbas BS, Sakati NA, Raef H, Al-ashwal AA (2005). Continuous subcutaneous insulin infusion in type 1 diabetic Saudi children. A comparison with conventional insulin therapy. Saudi Med J.

[15] Colino Alcol E, Lopez Capape M, Alvarez Gomez MA, Alonso Blanco M, Martin Frias M, Barrio Castellanos R (2006). Continuous subcutaneous insulin infusion in pediatric patients with type 1 diabetes mellitus. An Pediatr (Barc).

[16] Reznik Y, Morera J, Rod A, Coffin C, Rousseau E, Lireux B, Joubert M (2010). Efficacy of continuous subcutaneous insulin infusion in type 2 diabetes mellitus: a survey on a cohort of 102 patients with prolonged follow-up. Diabetes Technol Ther.

[17] Rothman KJ (1990). No adjustments needed for multiple comparisons. Epidemiology.

[18] Streiner DL, Norman GR (2011). Correction for multiple testing: Is there a resolution. Chest.

[19] Cooke DW, Plotnick L (2008). Type 1 diabetes mellitus in pediatrics. Pediatr Rev.

[20] Jeitler K, Horvath K, Berghold A, Gratzer TW, Neeser K, Pieber TR, Siebenhofer A (2008). Continuous subcutaneous insulin infusion versus multiple daily insulin injections in patients with diabetes mellitus: systematic review and meta-analysis. Diabetologia.

[21] Fuld K, Conrad B, Buckingham B, Wilson DM (2010). Insulin pumps in young children. Diabetes Technol Ther.

[22] Plotnick LP, Clark LM, Brancati FL, Erlinger T (2003). Safety and effectiveness of insulin pump therapy in children and adolescents with type 1 diabetes. Diabetes Care.

